# KDM2B variants in the CxxC domain impair its DNA-binding ability and cause a distinct neurodevelopmental syndrome

**DOI:** 10.1093/hmg/ddaf082

**Published:** 2025-05-27

**Authors:** Amber S E van Oirsouw, Michael A Hadders, Martijn Koetsier, Edith D J Peters, Nurit Assia Batzir, Tahsin Stefan Barakat, Diana Baralle, Adelyn Beil, Marie-Noëlle Bonnet-Dupeyron, Philip M Boone, Arjan Bouman, Deanna Alexis Carere, Benjamin Cogne, Leslie Dunnington, Laura S Farach, Casie A Genetti, Bertrand Isidor, Louis Januel, Aakash Joshi, Nayana Lahiri, Kristen N Lee, Idit Maya, Meriel McEntagart, Hope Northrup, Mathilde Pujalte, Kate Richardson, Susan Walker, Bobby P C Koeleman, Mariëlle Alders, Richard H van Jaarsveld, Renske Oegema

**Affiliations:** Graduate School of Life Sciences, Utrecht University, Heidelberglaan 8, 3584 CS Utrecht, The Netherlands; UMC Utrecht Brain Center, University Medical Center Utrecht, Utrecht University, Heidelberglaan 100, Utrecht 3584 CX, The Netherlands; Department of Genetics, University Medical Center Utrecht, Utrecht University, Heidelberglaan 100, Utrecht 3584 CX, The Netherlands; Oncode Institute and Center for Molecular Medicine, University Medical Center Utrecht, Utrecht University, Universiteitsweg 100, Utrecht 3584 CG, The Netherlands; Department of Genetics, University Medical Center Utrecht, Utrecht University, Heidelberglaan 100, Utrecht 3584 CX, The Netherlands; Department of Genetics, University Medical Center Utrecht, Utrecht University, Heidelberglaan 100, Utrecht 3584 CX, The Netherlands; Pediatric Genetics Unit, Schneider Children's Medical Centerl, 14 Kaplan St., Petah Tikva 4920235, Israel; Department of Clinical Genetics, Erasmus MC University Medical Center, Dr. Molewaterplein 50, Rotterdam 3000 CA, The Netherlands; Human Development and Health, Faculty of Medicine, University of Southampton, Southampton General Hospital, Tremona Road, Southampton SO16 6YD, United Kingdom; Department of Pediatrics, Division of Pediatric Genetics, Metabolism, and Genomic Medicine, University of Michigan, 1500 E. Medical Center Drive, Ann Arbor, MI 48109, United States; Consultations de Génétique, Centre Hospitalier de Valence, 179 Bd Maréchal Juin, Valence 26000, France; Division of Genetics and Genomics, Boston Children's Hospital, 300 Longwood Ave., Boston, MA 02115, United States; Department of Clinical Genetics, Erasmus MC University Medical Center, Dr. Molewaterplein 50, Rotterdam 3000 CA, The Netherlands; GeneDx, LLC, 207 Perry Parkway, Gaithersburg, MD 20877, United States; Nantes Université, Centre Hospitalier Universitaire de Nantes, Service de Génétique médicale 1, place Alexis Ricordeau, 44093 Nantes, France; Department of Pediatrics, Division of Medical Genetics, McGovern Medical School at the University of Texas Health Science Center at Houston (UTHealth Houston) and Children’s Memorial Hermann Hospital, 6431 Fannin Street, MSB 3144B, Houston, TX 77030, United States; Department of Pediatrics, Division of Medical Genetics, McGovern Medical School at the University of Texas Health Science Center at Houston (UTHealth Houston) and Children’s Memorial Hermann Hospital, 6431 Fannin Street, MSB 3144B, Houston, TX 77030, United States; Division of Genetics and Genomics, Boston Children's Hospital, 300 Longwood Ave., Boston, MA 02115, United States; Manton Center for Orphan Disease Research, Boston Children's Hospital, 300 Longwood Ave., Boston, MA 02115, United States; Nantes Université, Centre Hospitalier Universitaire de Nantes, Service de Génétique médicale 1, place Alexis Ricordeau, 44093 Nantes, France; Department of Genetics, Hospices Civils de Lyon, 3 Quai des Célestins, Lyon 69002, France; St. George’s University of London & St. George’s University Hospitals NHS Foundation Trust, Blackshaw Road, Tooting, London SW17 0QT, United Kingdom; St. George’s University of London & St. George’s University Hospitals NHS Foundation Trust, Blackshaw Road, Tooting, London SW17 0QT, United Kingdom; Department of Pediatrics, Division of Pediatric Genetics, Metabolism, and Genomic Medicine, University of Michigan, 1500 E. Medical Center Drive, Ann Arbor, MI 48109, United States; Recanati Genetics Institute, Beilinson Hospital, Rabin Medical Center, 39 Jabotinski St., Petah Tikva 49100, Israel; Gray Faculty of Medical and Health Sciences, Tel Aviv University, 35 Klatzkin St, Tel Aviv 6997801, Israel; St. George’s University of London & St. George’s University Hospitals NHS Foundation Trust, Blackshaw Road, Tooting, London SW17 0QT, United Kingdom; Department of Pediatrics, Division of Medical Genetics, McGovern Medical School at the University of Texas Health Science Center at Houston (UTHealth Houston) and Children’s Memorial Hermann Hospital, 6431 Fannin Street, MSB 3144B, Houston, TX 77030, United States; Department of Genetics, Hospices Civils de Lyon, 3 Quai des Célestins, Lyon 69002, France; Department of Pediatrics, Division of Medical Genetics, McGovern Medical School at the University of Texas Health Science Center at Houston (UTHealth Houston) and Children’s Memorial Hermann Hospital, 6431 Fannin Street, MSB 3144B, Houston, TX 77030, United States; Genomics England, Translational Genomics, One Canada Square, London E14 5AB, United Kingdom; UMC Utrecht Brain Center, University Medical Center Utrecht, Utrecht University, Heidelberglaan 100, Utrecht 3584 CX, The Netherlands; Department of Human Genetics, Amsterdam UMC, University of Amsterdam, Meibergdreef 9, Amsterdam 1105 AZ, The Netherlands; Reproduction and Development Research Institute, Meibergdreef 9, Amsterdam 1105 AZ, The Netherlands; Department of Genetics, University Medical Center Utrecht, Utrecht University, Heidelberglaan 100, Utrecht 3584 CX, The Netherlands; Department of Genetics, University Medical Center Utrecht, Utrecht University, Heidelberglaan 100, Utrecht 3584 CX, The Netherlands

**Keywords:** intellectual disability, congenital heart disease, chromatinopathy, clinical genetics, developmental delay

## Abstract

Rare variants affecting the epigenetic regulator KDM2B cause a recently delineated neurodevelopmental disorder. Interestingly, we previously identified both a general *KDM2B*-associated episignature and a subsignature specific to variants in the DNA-binding CxxC domain. In light of the existence of a distinct subsignature, we set out to determine if *KDM2B* CxxC variants are associated with a unique phenotype and disease mechanism. We recruited individuals with heterozygous CxxC variants and assessed the variants’ effect on protein expression and DNA-binding ability. We analyzed clinical data from 19 individuals, including ten previously undescribed individuals with seven novel CxxC variants. The core phenotype of the *KDM2B*-CxxC cohort is more extensive as compared to that of individuals with *KDM2B* haploinsufficiency. All individuals with CxxC variants presented with developmental delay, mainly in the speech and motor domain, in addition to variable intellectual disability and mild facial dysmorphism. Congenital heart defects were observed in up to 78% of individuals, with additional common findings including musculoskeletal, ophthalmological, and urogenital anomalies, as well as behavioral challenges and feeding difficulties. Functional assays revealed that while mutant KDM2B protein with CxxC variants can be expressed *in vitro*, its DNA-binding ability is significantly reduced compared to wildtype. This study shows that *KDM2B* CxxC variants cause a distinct neurodevelopmental syndrome, possibly through a molecular mechanism different from haploinsufficiency.

## Introduction

Pathogenic variants in genes involved in epigenetic regulation are an emerging cause of neurodevelopmental disorders (NDDs) [1]. We previously identified a novel NDD caused by heterozygous pathogenic variants in *KDM2B* and reported its associated episignature [2]. *KDM2B* encodes the 1336 amino acid lysine-demethylase 2B (KDM2B; OMIM 609078), which demethylates histone lysines K4, K36 and K79 via its JmjC domain [3–5], binds unmethylated CpG dinucleotides through its CxxC domain [6, 7] and interacts with other proteins through its F-box domain and leucine-rich region (LRR) [8, 9]. Apart from the canonical form of KDM2B (KDM2B-LF), a short isoform lacking the demethylating JmjC domain (KDM2B-SF) is expressed during early murine development and differentiation [8, 10].

KDM2B is implicated in many biological and cellular processes by regulation of gene expression, including cell proliferation [3, 11, 12] and differentiation [8, 13, 14]. Through its CxxC domain, KMD2B binds genome-wide to unmethylated CpG islands [7, 14]. In contrast to the majority of CpG dinucleotides that are methylated, CpG islands are short DNA regions enriched with unmethylated CpG dinucleotides. These CpG islands are therefore in a more permissive chromatin state and associated with approximately 70% of mammalian gene promoters [15].

Furthermore, KDM2B is essential for recruiting critical binding partners to CpG islands, such as Polycomb Repressive Complex 1 (PRC1) [7, 14]. Through its LRR, KDM2B interacts with core components of the PRC1, forming a non-canonical PRC1 variant 1 (PRC1.1). Polycomb group proteins play a critical role in repressing lineage-specific genes, thereby maintaining the undifferentiated state of embryonic stem cells [16]. Loss of KDM2B leads to ectopic *de novo* hypermethylation and silencing of PRC target genes and differentiation defects in mouse embryonic stem cells [13, 14]. Consistent with its pivotal role in differentiation, various mouse models have demonstrated the importance of KDM2B during early embryonic development [10, 13, 17–20].

Chromosomal deletions encompassing the 12q24.31 region including *KDM2B*, as well as putative loss-of-function (pLOF) variants, missense variants and a small in-frame deletion, have been identified in individuals presenting with a variable expression of disease including developmental delay (DD)/intellectual disability (ID), autism, attention deficit hyperactivity disorder (ADHD), congenital organ anomalies and facial dysmorphism [2, 21–26]. In our recently described cohort, 9 of 21 individuals had a variant located within the CxxC domain, suggestive of a mutational hotspot. In all nine individuals, the variants occurred *de novo* and congenital anomalies seemed overrepresented. Notably, DNA samples from this CxxC subgroup showed a distinct sub-signature, apart from the general KDM2B-associated episignature. This CxxC sub-signature exhibits an on average increased methylation level, even exceeding that of the hypermethylated probes of the general-KDM2B episignature [2].

We set out to characterize the *KDM2B* CxxC-disorder and its underlying molecular mechanism. We provide extensive clinical descriptions of ten previously undescribed individuals with seven novel variants, expanding the total cohort to 19 individuals. Furthermore, we performed functional analysis of CxxC variants to assess their effects on DNA-binding. Our findings suggest *KDM2B* CxxC variants are associated with a distinct neurodevelopmental disorder possibly through a dominant-negative mechanism as opposed to haploinsufficiency associated with loss-of-function variants.

## Results

### Individuals with CxxC variants

**Figure 1:**
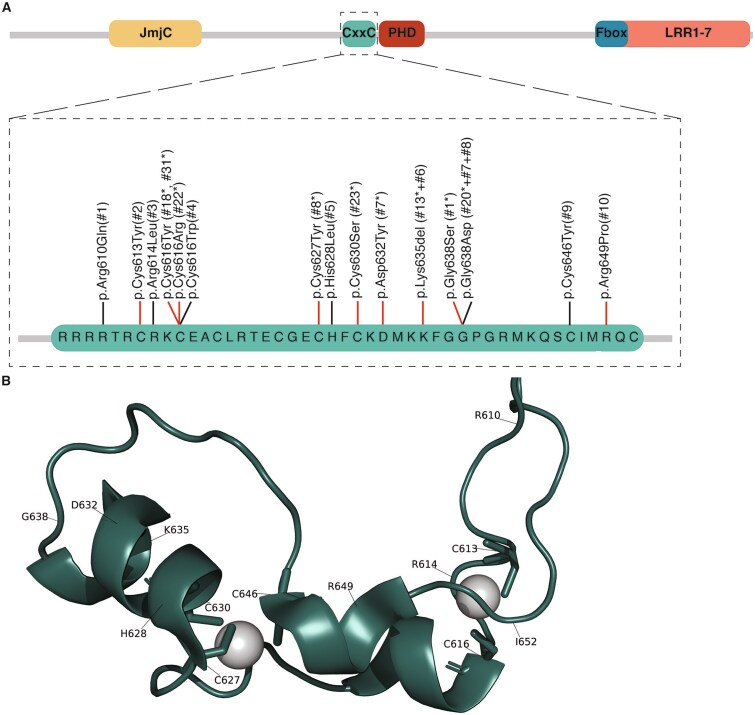
*KDM2B* CxxC variants. (A) Linear representation of the full-length KDM2B protein, depicting the functional domains JmjC, CxxC, PHD, F-box and LRR (Uniprot Q8NHM5). The CxxC domain is enlarged to amino acid level to show the location of the CxxC variants. All samples tested were positive for the KDM2B episignature (indicated with red bars: p.Cys613Tyr, p.Cys616Tyr, p.Cys616Arg, p.Cys627Tyr, p.Cys630Ser, p.Asp632Tyr, p.Lys635del, p.Gly638Ser, p.Arg649Pro). Previously published individuals are indicated with the individual numbering from the previous publication and an asterisk. (B) The 3D structure of KDM2B CxxC domain (PDB: 4O64 [6]) highlighting the position of the CxxC variants. The two zinc ions are shown as balls.

We identified ten unrelated individuals with nine distinct variants located in *KDM2B*’s CxxC domain (Table 1, Fig. 1A). All of the identified variants are missense variants, except for a single residue deletion p.(Lys635del) in individual #6. Interestingly, we observed p.(Lys635del) and p.(Gly638Asp) previously in unrelated individuals [2]. Clinical reports for each case are provided below, with a summary presented in Table 1.

**Table 1:** Clinical and genetic data of ten previously undescribed individuals with *KDM2B* CxxC variants. More extensive data are presented in Supplemental Table S1.

	**#1**	**#2**	**#3**	**#4**	**#5**	**#6**	**#7**	**#8**	**#9**	**#10**
**Sex, age**	F, 7y	M, 21y	M, 15y	F, 3y5m	M, 1y	F, 3y3m	M, 2y6m	M, 1y5m	M, 1y8m	M, 4y
**KDM2B variant**	c.1829G>A, p.(Arg610Gln)	c.1838G>A, p.(Cys613Tyr)	c.1841G>T, p.(Arg614Leu)	c.1848C>G, p.(Cys616Trp)	c.1883A>T, p.(His628Leu)	c.1903_1905del, p.(Lys635del)	c.1913G>A, p.(Gly638Asp)	c.1913G>A, p.(Gly638Asp)	c.1937G>A, p.(Cys646Tyr)	c.1946G>C, p.(Arg649Pro)
**Inheritance**	*De novo*	*De novo*	*De novo*	*De novo*	Inherited	*De novo*	*De novo*	*De novo*	*De novo*	*De novo*
**Development**										
**GDD (HP:0001263)**	No	Yes, mild	Yes, severe	Yes, moderate	Yes	Yes, mild	Yes	Yes	Yes	Yes
**ID (HP:0001249)**	Yes	Yes, mild	Yes, severe	UK	UK	UK	UK	UK	UK	Yes, moderate
**Motor delay (HP:0001270)**	No	Yes, mild	Yes	Yes	Yes	Yes, mild	Yes	Yes	Yes	Yes
**Speech/language delay (HP:0000750)**	Yes	Yes, mild	Yes	Yes	No	Yes, mild	Yes	Yes, mild	Yes	Yes
**Behavioural problems**	Autism	No	Autism, ADHD, self-mutilation, anxiety	No	UK	No	No	No	UK	Suspicion of autism, temper tantrums
**Growth**										
**OFC (SD)**	2.1	UK	−2.0 < SD < -1.3	−1.0	−0.8	−1.5	2.6	−0.2	−2	−1.3
**Height (SD)**	0.7	−1.5	< −2.7	0.7	−1.8	−0.2	0.4	−0.2	0.0	−0.7
**Weight to height (SD)**	1.5	0.9	−2.0 < SD < -1.3	0.0	UK	−0.2	0.7	−1.6	−1	−0.7
**Congenital anomalies**										
**Cardiovascular**	ASD II, atrial septal aneurysm, small VSD, ↓ ventricular function, mild PPS	No	Heart murmur, innocent	No	No	Aneurysmal atrial septum with PFO (at day two of life), small PDA	ASD, PDA, PFO, VSD, ventricular dilatation and bicuspid aortic valve	ASD, VSD	ASD	ASD II
**Urogenital**	No	No	No	No	No	No	Renal cysts, asymetric kidneys	Single kidney	No	Cryptorchidism
**Ophthalmological**	No	Nasolacrimal duct stenosis, myopia −1.5 dpt ODS	No	Strabismus, myopia, astigmatism	No	Intermittent exotropia with hypertropia and myopic astigmatism	Macrocornea	No	No	Duane anomaly of the right eye

*(continued)*

**Table 1:** Continued.

	**#1**	**#2**	**#3**	**#4**	**#5**	**#6**	**#7**	**#8**	**#9**	**#10**
**Other**										
**Facial dysmorphism**	Prominent cupid’s bow	Small ears with broad and folded helix, high anterior hairline, deepset eyes, and a relatively long shaped face. Bifid uvula	Simple ears with fleshy lobes	Arched eyebrows, downslanted palpebral fissures, midface retrusion, high columnella, broad nares, and large anteverted ears	High forehead, a triangular face short flared eyebrows, deep-set eyes, high nasal bridge, small nasal ridge, pointed upper lip, asymmetry of the ears	Flat occiput and top of head, scleral show, mild hypertelorism, prominent and laterally extending eyebrows, prominent nasal tip, ear creases on lobes originate at intertragal notch, retrognathia	Macroglossia (present since birth), tall forehead, infraorbital creases, midface retrusion	Right ear low set and posteriorly rotated, horizontal antihelix (R > L), mild micrognathia	No	Medial flaring of eyebrows, full upper eyelids, everted lower lip, overfolded helices, prominent antihelices
**Skeletal and limbs**	No	Pes planovalgus, tapering fingers, joint stiffness	Perthes disease, 5th finger clinodactyly, deviated halluces	Pes planovalgus	Mild left foot arthrogryposis and hip dysplasia	Sandal gap	Leg length discrepancy	Sandal gap	Bilateral feet postaxial hexadactyly	Pes planovalgus, valgus deformity of the knees, pectus carinatum
**Hypotonia (HP:0001252)**	No	No	No	Yes	Yes	Yes, mild	Yes, uses ankle foot orthosis	Yes	UK	No
**Feeding difficulties (HP:0011968)**	No	No	Yes	No	Yes	Yes	Picky eater	Yes	UK	UK
**Seizures (HP:0001250)**	No	No	No	No	UK	No	Yes	No	Yes	No
**Brain imaging**	NA	NA	UK	Normal	Arachnoid cysts	NA	Hydrocephalus/ventriculomegaly, posterior fossa cyst	Prominence of extra axial fluid spaces/supratentorial ventricles	Arachnoid cyst	NA

Abbreviations: ADHD, attention deficit hyperactivity disorder; ASD, atrial septal defect; dpt, diopters; GDD, global developmental delay; F, female; ID, intellectual disability; M, male; NA, not assessed; ODS, oculus dexter et sinister; OFC, occipital frontal circumference; PDA, patent ductus arteriosus; PFO, patent foramen ovale; PPS, peripheral pulmonary stenosis; SD, standard deviation; UK, unknown; VSD, ventricular septal defect; VUS, variant of uncertain significance.

### Clinical reports

Individual #1 (*de novo* c.1829G>A p.(Arg610Gln)) is a 7-year-old girl. At 6 weeks old, echocardiogram revealed a 5 mm secundum atrial septal defect (ASD), atrial septal aneurysm, tiny apical muscular ventral septal defect (VSD), and borderline mildly decreased left ventricular systolic function. She underwent an ASD closure at 2 years 4 months old. Gross motor milestones were achieved on time, but speech was delayed and speech and occupational therapy were initiated at 8 months old. First words were achieved at one year old. At her current age, she can say four-to-five-word sentences. Around 3 years old, concerns of autistic behaviors were raised and she was formally diagnosed with autism spectrum disorder at 5 years old. Since then, she has been attending applied behavioral analysis therapy, speech therapy, occupational therapy, and life skill classes. Her older sister had an ASD that spontaneously resolved. Dysmorphology exam revealed macrocephaly, Cupid’s bow upper lip and clubbing of toes bilaterally.

Individual #2 (*de novo* c.1838G>A p.(Cys613Tyr)) is a 21-year-old male with global developmental delay and mild ID. He spoke his first words and walked independently at 2.5 years old. He was diagnosed with bilateral absence of the nasolacrimal duct requiring surgery and mild myopia. He complains of joint stiffness. Facial features comprised a high anterior hairline, a relatively long face, deep-set eyes, and small ears with broad and overfolded helices. He was noted to have a bifid uvula, tapering fingers and pes planovalgus.

Individual #3 (*de novo* c.1841G>T p.(Arg614Leu)) is a 15-year-old boy. He has severe ID and behavioral challenges including autism spectrum disorder, ADHD, anxiety and self-harming behavior including skin picking. He can speak simple sentences since age 10 years. He was born prematurely after 35 weeks’ gestation and had feeding difficulties in infancy. He exhibited toe walking. His medical history includes constipation, Perthes disease, osteomyelitis and an episode of sepsis. On physical examination short stature was noted and below-average measurements for weight and head circumference. He has simple ears with fleshy earlobes, fifth finger clinodactyly and medially deviated halluces.

**Figure 2:**
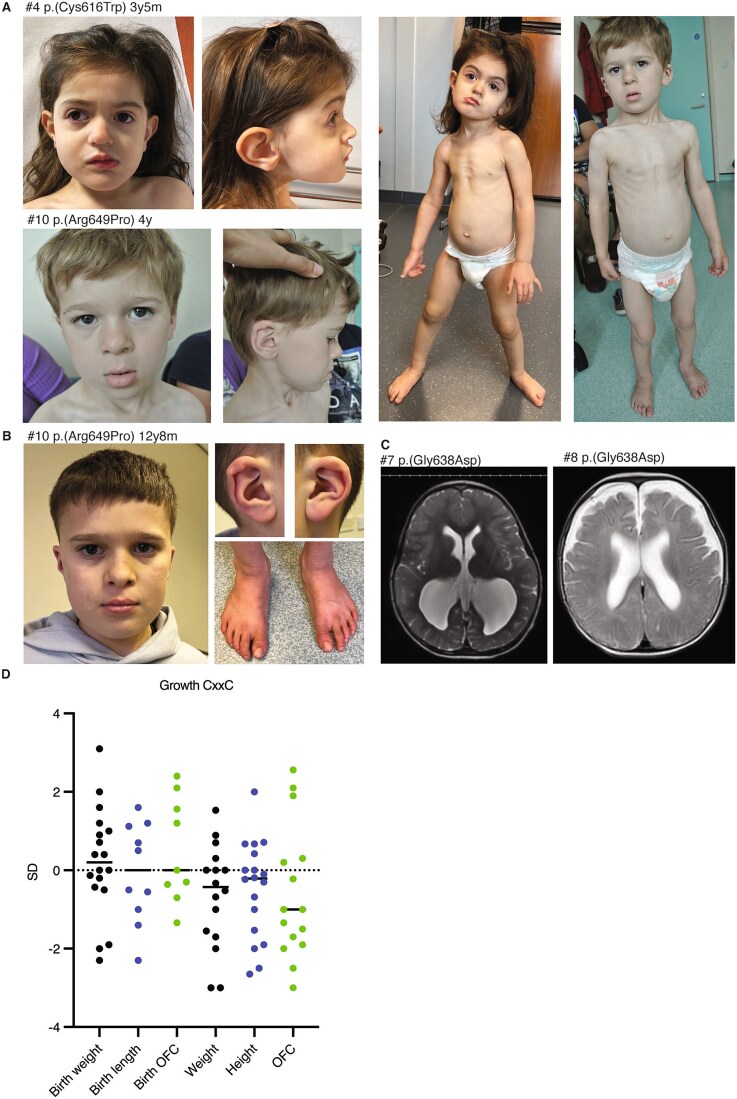
Photographs and growth data of individuals with *KDM2B* CxxC variants. (A) Photographs of individuals #4 and #10 at 3y5m and 4y, respectively. Individual #4 shows arched eyebrows, downslanted palpebral fissures, midface retrusion, high columnella, broad nares, large anteverted ears and pes planovalgus. Individual #10 exhibits medial flaring of the eyebrows, full upper eyelids, everted lower lip, overfolded helices, prominent antihelices, pectus carinatum, and pes planovalgus. (B) Photographs of individual #10 at 12y8m. (C) Brain axial magnetic resonance imaging of individual #7 and #8 showing enlarged lateral ventricles. (D) Growth measurements at last investigation of individuals with CxxC variants, expressed in standard deviations (SD), reveal a trend towards below-average postnatal growth.

Individual #4 (*de novo* c.1848C>G p.(Cys616Trp)) is a now 5-year-old girl with global DD, who was evaluated at 3.5 years old. She was born at 41 weeks by vaginal delivery with biometry in normal range. She began sitting at 23 months and walking at 28 months. Speech development was delayed with few words without association at 3 years old and speaking in short sentences with pronunciation difficulties at 5 years old. On physical examination she had arched eyebrows, downslanted palpebral fissures, midface retrusion, high columnella, broad nares, and large anteverted ears (Fig. 2A, S1A). She has visual impairment with strabismus, myopia and astigmatism. She has pes planovalgus requiring orthopedic shoes.

Individual #5 (paternally inherited variant of unknown significance (VUS) c.1883A>T p.(His628Leu)) was born at almost 40 weeks’ gestation with a low birth weight. He was admitted to the NICU for 5 days due to relatively mild respiratory problems. As a neonate he had gastro-esophageal reflux and diarrhea thought to be due to lactose intolerance. He had delayed motor development—at nearly 1 year old he was not sitting up and had significant head lag. Brain imaging showed a 25 mm diameter arachnoid cyst which was surgically removed. At 3 years old global muscle weakness was noted and significant motor delay was present at age 4 years. On examination (Fig. S1C) he has a high forehead, a triangular face, short flared eyebrows, deep-set eyes, high nasal bridge, small nasal ridge, pointed upper lip, asymmetry of the ears, and mild left foot arthrogryposis. He was also diagnosed with hip dysplasia.

Individual #6 (*de novo* c.1903_1905delAAG, p.(Lys635del)) is a 3-year-old girl who presented with hypotonia. She was born at term after an uncomplicated pregnancy with normal weight and length measurements. Excess neck skin was noted. Echocardiogram on day two of life showed a small patent ductus arteriosus (PDA) and aneurysmal atrial septum with patent foramen ovale (PFO). The neonatal period was significant for laryngomalacia, feeding issues and deformational plagiocephaly. She could sit independently at 7 months, stand with support at 11 months, and say two words by 11 months. Now at 3 years old, her core strength is improving, however she has expressive speech delay. On physical examination flat occiput, scleral show, prominent laterally extending eyebrows, mild hypertelorism, prominent nasal tip, creases on the ear lobes that originate at the intertragal notch, and retrognathia were noted. A hemangioma in the xiphoid area was noted. There is a wide angle between first and second toes bilaterally.

Individual #7 (*de novo* c.1913G> A, p.(Gly638Asp)) is a now 5-year-old boy with DD, who was evaluated at age 2.5 years. He was born at term with normal length and weight and large occipital-frontal circumference. He was diagnosed with neonatal hypolgycemias, congenital heart defects (bicuspid aortic valve, VSD, ASD, PFO, PDA, and ventricular dilatation), renal cysts with asymmetric kidneys, macrocornea and has shunted ventriculomegaly (Fig. 2C). Physical examination noted umbilical hernia, leg length discrepancy, midface retrusion, tall forehead, infraorbital creases and macroglossia.

Currently he is in preschool with children of one year younger, where he is learning well and can identify numbers up to 40. He is gentle and gets along well with his classmates. Speech development is still delayed with speaking in 4-6 word phrases. His gross and fine motor development was delayed with first steps after age 2 years, but this improved significantly. He has hypotonia and uses ankle foot orthosis. He is a picky eater.

Individual #8 (*de novo* c.1913G>A p.(Gly638Asp)) is a 17-month-old male. He was born at term with normal measurements. He has had failure to thrive and feeding difficulties temporarily necessitating a gastric tube. He was diagnosed with a VSD and ASD postnatally due to a murmur. He underwent surgical repair of his VSD and primary closure of the secundum ASD. Additionally, he has a single kidney. He has global DD, babbles and speaks some words. Brain imaging at 7 months showed mild prominence of extra-axial fluid spaces and supratentorial ventricles (Fig. 2C). On physical examination hypotonia and spasticity were noted, as well as a low-set and posteriorly rotated right ear with a horizontal antihelix, mild micrognathia, and a sandal gap.

Individual #9 (*de novo* c.1937G>A p.(Cys646Tyr)) is a 1-year-old boy who presented with congenital anomalies and DD. He was born at term after an uncomplicated pregnancy. He was diagnosed with an ASD (surgery will be required) and bilateral postaxial polydactyly of the feet. He has focal epilepsy which started at 1 month and was treated with valproate sodium. Brain imaging showed an arachnoid cyst. His development was delayed (sat at 9 m and walked at 18 m) with speech delay requiring speech therapy.

Individual #10 (*de novo* c.1946G>C p.(Arg649Pro)) is a now 12-year-old boy who was evaluated at age 4 years. He had gross motor delay. He now only has mild fine motor difficulties. His speech development was also severely delayed, with a few words at age 4 years, and now speaks in sentences but can still mix up words and strangers have difficulty understanding him. He has had temper tantrums and still has some difficulty expressing emotions but is socializing well. He has moderate intellectual impairment and is currently attending a special school. He has had surgery for a large secundum ASD and bilateral undescended testes and was diagnosed with a Duane anomaly of the right eye. Of note, tooth eruption was delayed and his baby teeth had to be surgically removed. On physical examination medial flaring of the eyebrows, full upper eyelids, everted lower lip, overfolded helices, prominent antihelices, pectus carinatum, and pes planovalgus were noted (Fig. 2A, 2B).

### The *KDM2B*-CxxC cohort

We analyzed the data of all 19 individuals with 15 distinct CxxC variants, including the ten individuals described here and the nine previously reported individuals (Table S1). All CxxC variants are of *de novo* origin, except for p.(His628Leu) which was inherited from an unaffected father in individual #5. As we were unable to test the paternal grandparents, postzygotic mosaicism in the father cannot be excluded. We obtained leucocyte-derived DNA samples from 1 published (#8*) and 3 novel individuals (#2, #6 and #10), which all tested positive for the general *KDM2B*-associated episignature (EpiSign v4). In total, all 9/19 cases with CxxC variants that underwent methylation profiling showed this signature (Fig. 1A, Table 2) [2]. All CxxC variants are classified as pathogenic according to the ACMG criteria (Table S2), except for variant p.(His628Leu). This variant is classified as a variant of unknown significance (VUS), due to paternal inheritance and lack of functional evidence supporting pathogenicity. Therefore, this individual was excluded from the clinical analysis of the CxxC cohort.

**Table 2:** Data of each CxxC variant, presence of the variant in gnomAD or RGC, summary of *in silico* prediction results, results from functional studies and ACMG classification. The ACMG variant scoring results are presented in Supplemenal Table S2.

	**R610Q**	**C613Y**	**R614L**	**C616R**	**C616Y**	**C616W**	**C627Y**	**H628L**	**C630S**	**D632Y**	**K635del**	**G638S**	**G638D**	**C646Y**	**R649P**
KDM2B variant	p.(Arg 610Gln)	p.(Cys 613Tyr)	p.(Arg 614Leu)	p.(Cys 616Arg)	p.(Cys 616Tyr)	p.(Cys 616Trp)	p.(Cys 627Tyr)	p.(His 628Leu)	p.(Cys 630Ser)	p.(Asp 632Tyr)	p.(Lys 635del)	p.(Gly 638Ser)	p.(Gly 638Asp)	p.(Cys 646Tyr)	p.(Arg 649Pro)
	c.1829 G>A	c.1838 G>A	c.1841 G>T	c.1846 T>C	c.1847 G>A	c.1848 C>G	c.1880 G>A	c.1883 A>T	c.1889 G>C	c.1894 G>T	c.1903_1905del	c.1912 G>A	c.1913 G>A	c.1937 G>A	c.1946 G>C
INH	*De novo*	*De novo*	*De novo*	*De novo*	*De novo*	*De novo*	*De novo*	Inherited	*De novo*	*De novo*	*De novo*	*De novo*	*De novo*	*De novo*	*De novo*
N =	1	1	1	1	2	1	1	1	1	1	2	1	3	1	1
**Population data**															
gnomAD/RGC	0x/0x	0x/0x	0x/0	0x/0x	0x/0x	0x/0x	0x/0x	0x/2x	0x/0x	0x/0x	0x/0x	0x/0x	0x/0x	0x/0x	0x/0x
***In silico* pathogenic prediction**															
AM, MT, SIFT, PP	4/4	4/4	4/4	4/4	4/4	4/4	4/4	4/4	4/4	4/4	2/2	4/4	4/4	4/4	4/4
Rosetta ΔΔG (n = 5, ±SD)	1.58 ±0.39 ↓DS	0.23 ± 1.48	−0.33 ± 0.30	1.65 ± 0.88 ↓DS	0.11 ± 1.32	0.25 ± 1.19	0.38 ± 0.45	0.26 ± 0.57	1.46 ± 0.43 ↓DS	2.77 ± 0.23 ↓DS	−0.03 ±0.04	4.37 ± 1.16 ↓DS	7.05 ± 0.65 ↓DS	7.40 ± 0.96 ↓DS	5.55 ± 0.99 ↓DS
Effect protein structure	Affect DNA binding	Affect zinc ion	Affect local protein structure	Affect zinc ion	Affect zinc ion	Affect zinc ion	Affect zinc ion	Affect local protein structure	Affect zinc ion	Affect local protein structure	Affect KFGG motif	Affect KFGG motif	Affect KFGG motif	Affect zinc ion	Affect DNA binding
**Functional studies**															
Episign	NT	Positive	NT	Positive	Positive	NT	Positive	NT	Positive	Positive	Positive	Positive	NT	NT	Positive
SF/LF (% of WT ± SD)	NT	92 ± 25/ 154 ± 31	NT	101 ± 31/126 ± 14	103 ± 35/142 ± 16	NT	107 ± 23/122 ± 21	NT	110 ± 26/129 ± 19	93 ± 18/ 126 ± 20	102 ± 11/157 ± 21	98 ± 15/ 137 ± 18	100 ± 22/142 ± 30	NT	NT
DNA binding	NT	NT	NT	↓	↓	NT	↓	NT	↓	↓	↓	↓	↓	NT	NT
**ACMG classification**															
	5	5	5	5	5	5	5	3	5	5	5	5	5	5	5

Abbreviations: AM, AlphaMissense; ↓DS, destabilizing; INH, inheritance; MT, MutationTaster; N, number of individuals; NT, not tested; PP, PolyPhen; ↓, significantly decreased (p < 0.0001).

All 18 individuals exhibit developmental delay in one or multiple domains, especially early speech (18/18) and motor delay (15/18) (Table 3, Table S1). Cognitive function ranged from below-average IQ to severe ID with absence of speech. Frequent features present in at least half of individuals include cardiovascular anomalies (14/18), particularly ASD (n = 11), single kidney (5/18), ophthalmological anomalies (10/18), skeletal anomalies (12/18), hypotonia (7/16), feeding difficulties (6/11) and behavioral challenges (8/18). Brain imaging revealed abnormalities in five individuals (5/10), with cysts and/or enlarged ventricles in three, for which one of these individuals underwent surgery. Growth measurements indicated a trend towards below-average growth (Fig. 2D). Microcephaly (4/15) and macrocephaly (2/15) were present in a minority. Facial dysmorphism was present in nearly all individuals (17/18). Analysis of available photographs (n = 7) revealed recurrent features, including downslanted palpebral fissures, a bulbous nasal tip, a prominent cupid’s bow of the upper lip, full cheeks, asymmetric or dysplastic ears and prominent ear lobes.

**Table 3:** Frequency of clinical features present in the CxxC cohort (n = 18).

**FEATURES**	**FREQUENCY (%)**
Development	
ID (HP:0001249)/learning difficulties	10/13 (77%)
Motor delay (HP:0001270)	15/18 (83%)
Speech delay (HP:0000750)	18/18 (100%)
Behavioral challenges	8/18 (44%)
Autism (HP:0000729)/autistic features	6/18 (33%)
ADHD (HP:0007018)	3/16 (19%)
Growth	
Microcephaly (HP:0000252)	4/15 (27%)
Macrocephaly (HP:0000256)	2/15 (13%)
Height ≤ −1 SD	6/18 (33%)
Height ≥ 1 SD	1/18 (6%)
Weight ≤ −1 SD	6/16 (38%)
Weight ≥ 1 SD	1/16 (6%)
Organ system anomalies	
Congenital heart defects	14/18 (78%)
ASD (HP:0001631)	11/18 (61%)
PFO (HP:0001655)	5/18 (28%)
VSD (HP:0001629)	4/18 (22%)
Pulmonary stenosis (HP:0034350)	4/18 (22%)
PDA (HP:0001643)	4/18 (22%)
Urogenital anomalies	8/18 (44%)
Single kidney (HP:0000122)	5/18 (28%)
Opthalmological anomalies	10/18 (56%)
Myopia (HP:0000545)	4/18 (22%)
Nasolacrimal duct stenosis (HP:0007678)	3/18 (17%)
Abnormal brain imaging	5/10 (50%)
Intracranial cysts	3/10 (30%)
Ventriculomegaly (HP:0002119)	3/10 (30%)
Other	
Facial dysmorphism	17/18 (94%)
Skeletal and limbs	12/18 (67%)
Pes planovalgus (HP:0001763)	5/18 (28%)
Hypotonia (HP:0001252)	7/16 (44%)
Feeding difficulties (HP:0011968)	6/11 (55%)
Seizures (HP:0001250)	2/18 (11%)

Abbreviations: ADHD, attention deficit hyperactivity disorder; ASD, atrial septal defect; ID, intellectual disability; PDA, patent ductus arteriosus; PFO, patent foramen ovale; VSD, ventricular septal defect.

Collectively, the core phenotype observed in the expanded CxxC cohort includes developmental delays, primarily affecting the speech and motor domain, along with variable intellectual deficits, behavioral challenges, congenital heart defects, urogenital anomalies, ophthalmological anomalies, musculoskeletal anomalies, feeding difficulties and facial dysmorphism. This phenotype is more extensive than that observed in individuals with other pathogenic non-CxxC missense or pLOF variants (n = 6) [2], who do not have congenital cardiovascular or ophthalmological anomalies nor kidney agenesis. Moreover, four individuals with a 12q24.31 microdeletion encompassing *KDM2B*, who exhibit the *KDM2B* episignature [2], do not present with cardiovascular, ophthalmological or urogenital anomalies [24–26]. These findings suggest that the distinct phenotypic features observed are specifically attributable to CxxC variants.

### *In silico* modeling of CxxC variants

To improve our understanding of how these CxxC variants lead to the described disorder, we first performed *in silico* assessment of all novel and published CxxC variants (n = 15). *In silico* prediction programs predict these variants to be pathogenic (Table 2) and all residues are intolerant to variation [27]. In accordance, all variants are absent from gnomAD [28] and RGC [29], except for p.(His628Leu), which is reported twice in RGC (Table 2) and is the variant inherited from an asymptomatic father. Seven of 15 variants, affecting residues Cys613, Cys616, Gly638 and Cys646, correspond to conserved positions within the homologous CxxC domain of KMT2A, where pathogenic mutations have been previously reported in patients with Wiedemann-Steiner syndrome [30, 31] (ClinVar Variation ID: 429638 Accession: VCV000429638.2).

The variants are located throughout the CxxC domain (Fig. 1B). The CxxC domain contains two conserved CXXCXXC motifs, where cysteine residues chelate a zinc ion, forming two zinc-fingers [6]. Half of the CxxC variants (7/15) result in the loss of a cysteine residue. A linker region after the two CXXCXXC motifs contains a highly conserved KFGG motif that is predicted to provide rigidity to the CxxC domain [32], and is mutated in 3/15 variants at positions 635 and 638. The KFGG motif is followed by a DNA-binding motif (KQ) that forms hydrogen bonds with the CpG dinucleotide in the DNA major groove. The DNA minor groove is bound by positively charged N-terminal and C-terminal regions of the CxxC domain [6, 32]. Specifically, Arg610 and Arg649 are predicted to form hydrogen bonds with the backbone of the DNA minor groove, as shown for the homologous residues in the CxxC domain of DNMT1 [33]. Additionally, Arg610 was found to form several hydrogen bonds with residues in the PHD domain [6]. Due to the rigidity of the CxxC domain by its KFGG motif and its proximity to the DNA during DNA-binding, methylation of cytosines prevents DNA binding by KDM2B’s CxxC domain [32]. No CxxC variants in our cohort affect the DNA-binding motif of the CxxC domain. However, the rigidity of the CxxC domain could be affected by variants in the KFGG motif, potentially influencing KDM2B’s DNA target sites. The variants at positions 614, 628 and 632 result in more hydrophobic residues, which can cause loss of hydrogen bonds and/or disturb correct folding. Specifically, Arg614 could be part of the positively charged residues that contribute to DNA binding, His628Leu alters the surface that could affect interaction or reduce solubility and Asp632Tyr alters the local protein surface, potentially affecting stability of the fold and binding partners. Taken together, the CxxC variants are predicted to affect zinc ion binding, DNA binding or the local protein structure (Table 2).

### Mutant KDM2B is expressed *in vitro*

As the patient variants affect highly conserved residues [2] important for local protein structure, we hypothesized they may influence protein stability. Protein stability changes were predicted *in silico* using the tool Rosetta ΔΔG, where positive ΔΔG values indicate destabilizing variants and negative values indicate stabilizing variants. Using a threshold of 1, half of the variants (p.(Arg610Gln), p.(Cys616Arg), p.(Cys630Ser), p.(Asp632Tyr), p.(Gly638Ser), p.(Gly638Asp), p.(Cys646Tyr), p.(Arg649Pro)) were predicted to destabilize the CxxC domain and none reached the threshold of a stabilizing effect (Table 2).

**Figure 3:**
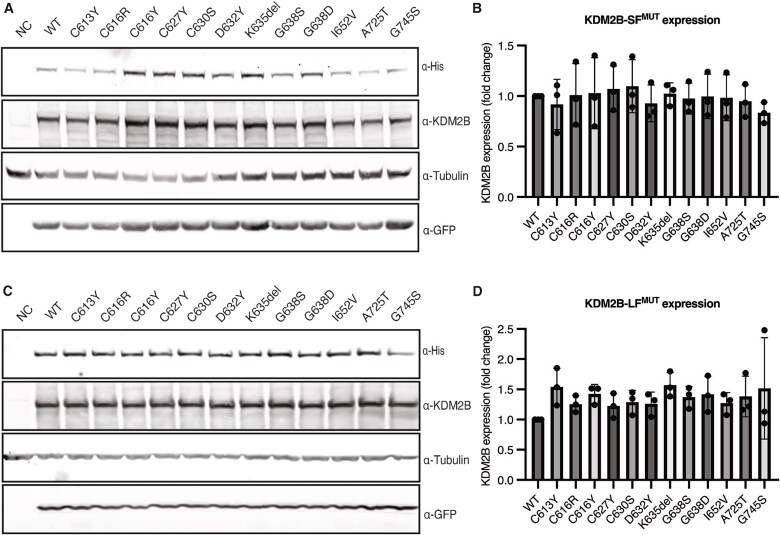
Expression of KDM2B short form (SF) and long form (LF). (A + C) Western blot results of wildtype (WT) and mutant his-KDM2B-SF (A) and mutant his-KDM2B-LF (C) expression in HEK293T cells following transfection with WT or mutant His-KDM2B expression plasmid. Tubulin was used as a loading control, GFP as transfection control. An untransfected sample was also included (NC). (B + D) Quantification of western blot results of KDM2B-SF (B) and KDM2B-LF (D) expression revealing all variants are expressed, similar to WT (n = 3 replicates). KDM2B expression was normalized to the transfection control GFP and WT KDM2B expression.

To evaluate whether KDM2B CxxC variants affect its expression, we compared protein expression of mutant KDM2B (KDM2B^MUT^) to wildtype (WT) KDM2B (KDM2B^WT^) by overexpression in HEK293T cells. We included nine CxxC variants observed in our cohort and three previously published VUS located near the CxxC domain (p.(Ile652Val), p.(Ala725Thr), p.(Gly745Ser)) [2, 21, 23]. All variants showed similar expression levels of both KDM2B-SF and KDM2B-LF as compared to wildtype (Fig. 3A-D, Fig. S2, Table 2). Recognizing that our overexpression system may not reflect the endogenous expression, our results suggest that while the variants may impact CxxC domain stability, they do not negatively affect expression of either KDM2B isoform.

### CxxC variants impair DNA-binding ability

**Figure 4:**
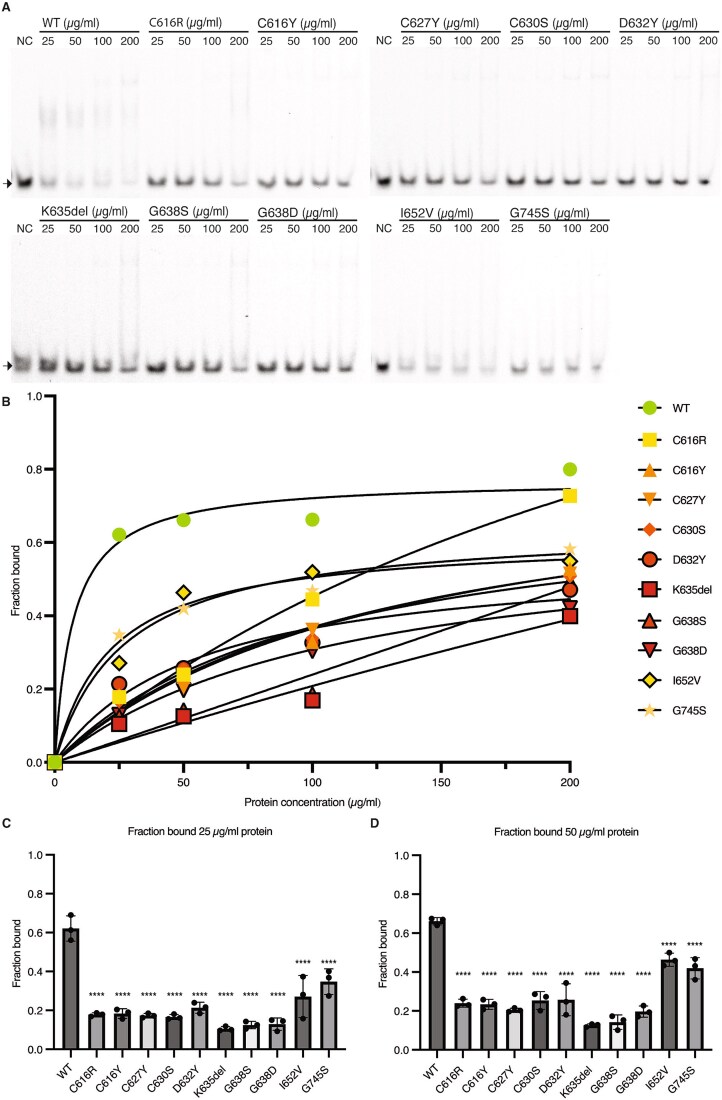
CxxC variants impair DNA-binding ability. (A) EMSA results of WT or mutant CxxC protein. WT or mutant CxxC protein was incubated in increasing amounts (25, 50, 100 and 200 μg/ml) with fluorescently labeled DNA probe containing unmethylated CpG dinucleotides. A sample without addition of protein was also included (NC). Arrows point to unbound CpG DNA. The DNA shift indicates CpG DNA is bound by protein. (B) Quantification of EMSA results (n = 3). Binding curves were obtained through fitting non-linear regression hyperbola. (C + D) Bar graphs of fraction bound of WT and mutant CxxC protein of 25 μg (C) and 50 μg (D). Statistical results of one-way ANOVA with Dunnett’s multiple comparisons test of mutant CxxC protein compared to WT CxxC protein are shown (ns = p > 0.05, * = p < 0.05, ** = p < 0.01, *** = p < 0.001, **** = p < 0.0001). Alt Text. Electrophoretic mobility shift assay results and quantification show that KDM2B CxxC variants reduce DNA-binding ability compared to wildtype, with statistical analysis confirming significantly impaired binding at specific protein concentrations.

Since mutant KDM2B protein with CxxC variants can be expressed in an *in vitro* overexpression system, we proceeded to investigate their effect on KDM2B’s DNA-binding ability. The CxxC domain of KDM2B specifically recognizes unmethylated CpG dinucleotides [6, 7], therefore we tested DNA binding with an electrophoretic mobility shift assay (EMSA) using unmethylated DNA probes containing two CpG islands. For this, we produced recombinant wildtype (CxxC^WT^) or mutant (CxxC^MUT^) KDM2B CxxC-PHD protein (amino acids 600-750) (Fig. S3), as previous reports indicated the CxxC domain requires its adjacent PHD domain for stability [6]. CxxC^WT^ showed DNA binding upon addition of 25 μg/ml protein, which minimally increased upon adding more protein, suggesting WT protein reached DNA-binding saturation (Fig. 4A, 4B). DNA binding was significantly decreased upon introduction of all CxxC variants (<30% bound protein, p < 0.0001) (Fig. 4B, 4C, Fig. S4). Increasing the concentration of CxxC^MUT^ resulted in a higher fraction of protein-bound DNA, but for all variants, except CxxC^C616R^, a reduction of at least 25% remained when compared to WT (Fig. 4D. Additionally, VUS p.(Ile652Val) and p.(Gly745Ser) affected DNA binding (27% and 35% bound protein, respectively, p < 0.0001), despite these variants being located on the edge and outside of the CxxC domain, respectively. We were unable to produce VUS p.(Ala725Thr), which purified as a truncated fragment, thereby precluding DNA binding assays. In conclusion, our data demonstrate the tested variants significantly reduce the DNA-binding ability of KDM2B’s CxxC domain *in vitro*.

## Discussion

In this study we analyse in detail the clinical findings and genetic characteristics of nine individuals with pathogenic variants in the CxxC domain of *KDM2B*. Combined analysis of this cohort together with our previously published cohort shows that these variants cause a distinct neurodevelopmental syndrome. The *KDM2B*-CxxC disorder is characterized by developmental delays, mainly in the speech and motor domain, variable intellectual disability, congenital heart defects and facial dysmorphism. Speech delay is universal and remained a significant problem throughout childhood for several individuals. We observed behavioral challenges, hypotonia, feeding difficulties, urogenital anomalies, ophthalmological anomalies and pes planovalgus in multiple individuals. Neuroimaging was abnormal in half of individuals for whom this was performed, and neurosurgical intervention due to hydrocephalus was necessary in one individual. Since individuals with other pathogenic missense and pLOF *KDM2B* variants [2] do not show congenital cardiac or ophthalmological anomalies nor kidney agenesis, these features appear to be distinct phenotypic characteristics specifically attributable to CxxC variants. Based on our findings, we recommend a baseline workup of cardiac and renal ultrasound in each *KDM2B*-CxxC patient, and early developmental support focusing on speech, behavior and motor development as appropriate. In addition, ophthalmological examination and brain imaging should be considered.

In line with the more extensive clinical phenotype compared to patients with *KDM2B* haploinsufficiency, our *in vitro* assays suggest CxxC variants act through a different, likely dominant-negative disease mechanism. We show CxxC variants do not negatively affect expression of the long and short KDM2B isoform in an *in vitro* overexpression system. However, we observed a significantly decreased DNA-binding ability of CxxC variants using EMSA. We hypothesize this decreased DNA binding impairs the recruitment of KDM2B’s binding partners to target DNA. In this way it could prevent PRC1 from exerting its repressive function, which can be detrimental as haploinsufficiency of other PRC1 components has already been associated with monogenic disease (e.g. oculofaciocardiodental syndrome caused by BCOR variants (MIM 300166), and Luo-Schoch-Yamamoto syndrome caused by RING1B/RNF2 variants (MIM 619460)).

We observed a decreased DNA-binding ability of CxxC variants with EMSA, in a simplified and controlled environment. This setup allows us to specifically and directly observe protein-DNA interactions, minimizing interference from other cellular components. In a nuclear environment, KDM2B has numerous target sites across the genome, and has been shown to bind genome-wide to ±84% of CpG islands in mouse embryonic stem cells (mESCs) [7, 14]. Additionally, in the cellular context, KDM2B faces competition from other DNA-binding factors. The DNA-binding saturation we reach for the wildtype protein *in vitro* is unlikely to fully reflect the dynamic cellular environment, therefore CxxC variants may even impact DNA binding more severely *in vivo*.

KDM2B requires the ability of its CxxC domain to bind unmethylated CpG islands for its various roles in epigenetic regulation. DNA binding by KDM2B plays a crucial role in protecting CpG islands from *de novo* methylation during early murine development [13, 18]. The loss of KDM2B at its target sites has been shown to result in hypermethylation at promoters normally bound by KDM2B in mESCs [13]. In line with these studies, we previously identified a stronger increase in DNA hypermethylation in our CxxC cohort as compared to the entire *KDM2B* cohort, contributing to a distinct episignature [2]. Taken together with the current findings that demonstrate CxxC variants significantly decrease KDM2B’s DNA-binding ability, this suggests that KDM2B is also important in protecting DNA from methylation during human development. Further research is required to assess the effect of this hypermethylation in patients and to identify the pathways involved.

Beyond regulating DNA methylation, the CxxC domain of KDM2B is also important for recruiting the PRC1 complex to the DNA, thereby leading to the repression of lineage-specific genes [7, 14, 17, 19, 20]. Loss of KDM2B and its CxxC domain results in the upregulation of polycomb repressed target genes and induces early differentiation in mESCs [7, 14]. This disruption affects embryonic development, as demonstrated in KDM2B-deficient mouse models. Complete loss of KDM2B is embryonically lethal, causing severe developmental abnormalities including smaller body size, failure of neural tube closure, limb and craniofacial malformations and absence of the heart and aorta [13, 34]. Interestingly, mice homozygous for a KDM2B allele lacking only the CxxC domain also exhibit embryonic lethality with severe developmental defects [17, 20]. Heterozygous loss of the CxxC domain results in partial lethality and surviving mice display severe skeletal malformations with homeotic transformations [17], while mice heterozygous for *KDM2B* are free of developmental defects [13, 34]. Similar phenotypes to CxxC-specific loss are observed in RING1A or RING1B knockout models, underscoring the connection to the PRC complex [35, 36]. Additionally, heterozygous conditional loss of the CxxC domain in neurons results in autism/ID-like behavior in mice, such as impaired spatial and motor learning, deficits in fear conditioning and social impairments [19, 20]. These phenotypes overlap with the neurodevelopmental abnormalities observed in our cohort. Future studies are necessary to fully understand how CxxC variants cause their specific phenotypic effect including congenital anomalies.

KDM2B expands the list of CxxC-containing proteins associated with germline variants causing human disorders. KMT2A encodes a histone lysine methyltransferase that plays a critical role in regulating gene expression during early development and hematopoiesis [37]. Heterozygous KMT2A variants cause Wiedemann-Steiner syndrome (MIM 605130), which is characterized by DD, ID, and characteristic facial features, with or without additional congenital anomalies. Missense variants in KMT2A’s CxxC DNA-binding domain may be associated with more significant neurodevelopmental issues [30]. In other genes containing CxxC domains—KMT2B associated with intellectual developmental disorder (MIM 619934) and dystonia (MIM 617284), DNMT1 associated with autosomal dominant cerebellar ataxia, deafness, and narcolepsy (MIM 604121) and with hereditary sensory neuropathy type IE (MIM 614116), TET3 associated with neurodevelopmental Beck-Fahrner syndrome (MIM 618798)—pathogenic variants specifically affecting their CxxC domains have not been reported.

In conclusion, we delineate *KDM2B*-CxxC disorder as a distinct epigenetic neurodevelopmental syndrome with congenital anomalies. This more extensive presentation of the *KDM2B* disorder suggests a distinct effect of CxxC variants, potentially caused by impaired binding of KDM2B to CpG dinucleotides.

## Materials and methods

### Patient inclusion and data collection

This study is part of an ongoing investigation into genotype–phenotype correlations in *KDM2B* syndrome, approved by the medical ethical committee installed by the University Medical Centre Utrecht (TCBIO 21-355, March 18, 2021). The cohort was established through international collaborations using the GeneMatcher platform [38], direct personal communication and analysis of the Genomics England cohort [39]. *KDM2B* variants of interest were identified in Genomics England [39] by filtering aggV2 for heterozygous *KDM2B* variants with a maximum allele frequency < 0.001 and a maximum frequency of 5 in gnomAD v4.1.0 [28]. Among 78 195 individuals, five individuals with a CxxC variant were identified. Their referring physicians were contacted, and three were successfully included in this study (individuals #3, #5 and #10).

Inclusion required the identification of a *KDM2B* variant (NM_032590.5) located within the CxxC domain. Clinical and genetic data were collected using a standardized spreadsheet and subsequently entered into an electronic data capture platform. Informed consent for publication was obtained from all participants or their legal representatives. Facial photographs were systematically assessed by an experienced dysmorphologist (RO).

The phenotype of the CxxC cohort was compared to individuals with pathogenic non-CxxC missense variants, pLOF variants and 12q24.31 deletions published in our initial cohort (individuals 3.2, 4.1, 4.2, 4.3, 6, 11, 25.1, 25.2, 29, 30). Individuals 3.1 and 10 were excluded from this analysis due to the presence of a second genetic diagnosis affecting their phenotypic presentation [2].

### Analysis of *KDM2B* variants

Leucocyte-derived DNA samples were collected and EpiSign testing for the general *KDM2B*-associated episignature was conducted as previously described [2]. Variant population frequencies and gene constraint scores were obtained from gnomAD v4.1.0 [28] and RGC v1.1.2 [29]. Multiple *in silico* prediction programs were consulted: Polymorphism Phenotyping v2 (PolyPhen) [40], Sorting Intolerant from Tolerant (SIFT) [41], AlphaMissense [42], MutationTaster [43] and Rosetta DDG [44]. Structural analysis of variants was performed using PyMOL (The PyMOL Molecular Graphics System, Version 2.5.5, Schrödinger, LLC) and PDB 4O64 [6]. Variants were classified according to the 2015 American College of Medical Genetics (ACMG) and Genomics and the Association for Molecular Pathology guidelines [45].

### DNA constructs

The recombinant ZF-CxxC construct encompassing human *KDM2B* (encoding amino acids 600–750) including an N-terminal 6-his tag followed by a tobacco etch virus (TEV) protease cleavage site was a kind gift of Neil Blackledge (previously described [7]).

The His-KDM2B-LF (ENSP00000366271 = NP_115979.3) and His-KDM2B-SF (XP_005254018.1) constructs were created with In-Fusion cloning (Takara 5X In-Fusion HD Enzyme Premix, Takara Bio), using GFP-FBXL10 (Addgene plasmid 126 542) and a backbone including a EF1A promoter, P2A-EGFP-T2A-PuroR and AmpR (previously described [46]). Agilent Quikchange II XL-Site directed mutagenesis kit (Agilent Technologies) was used to introduce all patient variants in the wildtype KDM2B constructs.

### Protein expression in HEK293T cells

Transfection of wildtype and mutant His-tagged KDM2B-SF and KDM2B-LF was performed in HEK293T cells (n = 3, Fig. 3, S2). HEK293T cells were grown in Dulbecco’s Modified Eagle’s Medium (DMEM)—high glucose (Sigma Aldrich) supplemented with 10% fetal bovine serum (Bodinco) and 1% penicillin–streptomycin (Gibco). Cells were incubated at 37°C in 5% CO_2_, 95% air in humidified cell culture incubators. HEK293T cells were seeded on day 1 in 12-wells plates at 30-40% confluency. The next day, cells were transiently transfected using 1 μg plasmid DNA (KDM2B-SF/LF WT or variants) with 3 μL PEI (1 mg/ml) (Sigma Aldrich) in 100 μL Opti-MEM (Gibco). On day 3, cells were harvested and lysed with 100 μL Pierce RIPA buffer (Thermo Scientific, 89 900), containing protease inhibitor (cOmplete Mini, EDTA-free protease inhibitor cocktail tablets, Roche, 11 836 170 001). Samples were sonicated 3x30 seconds at 4°C at a low frequency with the BioRuptor Pico. SDS-PAGE was performed using Mini Gel Tank from Invitrogen Thermofisher, with a NuPage 4-12% Bis tris gel (1 mm x 15 well) (Invitrogen) for KDM2B-SF or a NuPage 3-8% Tris-Acetate gel (Invitrogen) for KDM2B-LF. Transfer was via a BIO-RAD Trans-Blot Turbo to a 0.2 μm nitrocellulose mini (BIO-RAD) at 25 V, 1.3A for 10 minutes. The following primary antibodies were used for KDM2B (Sigma-Aldrich 09-864, 1:1000), GFP (Sigma SAB4301138, 1:5000), 6x-His-tag (Invitrogen MA1-21315, 1:2000), α-tubulin (Invitrogen, 1:2000), with secondary antibodies Goat anti-Rabbit IRDye® 800CW (LICOR 926-32 211, 1:7500) and Goat anti-Mouse Alexa Fluor 680 (Invitrogen A21057, 1:7500). The blots were scanned with the Amersham Typhoon scanner.

### Expression and purification of recombinant KDM2B ZF-CxxC

We purified wildtype and variant-carrying recombinant KDM2B CxxC-PHD protein from bacteria (Fig. S3). Recombinant KDM2B ZF-CxxC constructs were expressed in Rosetta (DE3) cells. Bacterial cultures (100 ml) were grown in auto-induction medium (TB with 100 μg/ml kanamycin, 0.25 mM zinc, 0.2% lactose, 0.05% glucose, 2 mM MgSO_4_) at 37°C for 2-3 hours, after which the cultures were incubated at room temperature overnight. Rosetta cells were pelleted and lysed in 20 mM Tris–HCl (pH 8.0), 500 mM NaCl, 0.1% Nonidet P-40, complete EDTA-free protease inhibitors (Roche Applied Science) by sonicating on output setting 80A at 50% for 1-1.25 minutes with a probe sonicator. Lysed samples were centrifuged at 4°C for 30 minutes at 14000 r.p.m. (rotor FA-45-30-11). The supernatant was loaded onto Cytiva HisTrap FF Crude columns pre-equilibrated with buffer A (20 mM Tris–HCl (pH 8.0), 300 mM NaCl, 10% glycerol, 20 mM imidazole). The columns were then attached to an Akta FPLC and washed with 50 column volumes of buffer A + 8% buffer B (20 mM Tris–HCl (pH 8.0), 300 mM NaCl, 10% glycerol, 250 mM imidazole). The recombinant KDM2B ZF-CxxC protein was then eluted from the column with 100% buffer B. The KDM2B-containing elution fractions were pooled and desalted to remove the imidazole (desalting size exclusion chromatography) using PD-10 desalting column packed with Sephadex G-25 resin (pre-equilibrated with desalt buffer 20 mM Tris–HCl (pH 8.0), 10 mM NaCl, 40 mM KCl, 10% glycerol, 1 mM DTT). Proteins were concentrated using centrifugal concentrators (Vivaspin® 6, 5000 MWCO PES) at 1972 g, 4°C. Protein concentration was estimated using absorbance at 280 nM. Protein expression and purification was checked by SDS-PAGE followed by InstantBlue staining (expedeon, ISB1L) or Western Blot (anti-His tag) (Fig. S3).

### EMSA

A short randomly generated DNA probe (GTAGGCGGTGCTACACGGTTCCTGAAGTG) containing two CpGs (also described by [47]) was ordered from IDT with 5′ ends labeled with Alexa Fluor 660 and resuspended in sodium chloride-Tris buffer (100 mM NaCl; 10 mM Tris–HCl, pH 8.0). EMSA reactions were assembled in binding buffer (10 mM Tris–HCl pH 7.5, 40 mM KCl, 10 mM NaCl, 1 mM MgCl2, 1 mM EDTA, 1 mM DTT, 25 ng/μl poly-dAdT competitor DNA, 5% glycerol) with 1.25 nM fluorescent probe. The mixture was allowed to incubate for 15-20 minutes at room temperature prior to loading onto a 5% polyacrylamide gel containing 0.5x TBE (45 mM Tris-Borate, 1 mM EDTA) and 2.5% glycerol, using a mini protein gel system with running buffer (0.5x TBE, 2.5% glycerol). Gel electrophoresis was performed at 80 V for 40 minutes, after which the gels were directly scanned on an Amersham Typhoon biomolecular imager.

### Analysis expression and EMSA

A python script was written and used to automatically analyze the Western blot and EMSA TIF files. Blobs were detected and removed using OpenCV. The average intensity along individual lanes was calculated and a background line was drawn based on background levels of these intensity curves.

For Western blot analysis, the area of the peak was determined using derivatives of this peak and their intersection with the background line. KDM2B expression was normalized to the transfection control GFP. KDM2B^MUT^ expression was calculated per Western blot relative to KDM2B^WT^ expression.

For EMSA, the total area under the curve (AUC) of the intensity curves per individual lanes was divided in a bound and an unbound area by creating derivatives of the intensity peak of unbound probe and using their intersection with the background line. The bound fraction was calculated by dividing the AUC consisting of bound probe by the total AUC. Binding curves were made in Prism 10, using a non-linear regression hyperbola. Statistical analysis was performed in Prism 10. One-Way ANOVA with Dunnett’s multiple comparisons test was used to analyze the bar graphs of bound protein fractions (patient variants compared to wildtype).

## Supplementary Material

Supplemental_figures_ddaf082

Supplemental_tables_ddaf082
